# The *GCR2* Gene Family Is Not Required for ABA Control of Seed Germination and Early Seedling Development in Arabidopsis

**DOI:** 10.1371/journal.pone.0002982

**Published:** 2008-08-20

**Authors:** Jianjun Guo, Qingning Zeng, Mohammad Emami, Brian E. Ellis, Jin-Gui Chen

**Affiliations:** 1 Department of Botany, University of British Columbia, Vancouver, British Columbia, Canada; 2 Michael Smith Laboratories, University of British Columbia, Vancouver, British Columbia, Canada; Purdue University, United States of America

## Abstract

**Background:**

The plant hormone abscisic acid (ABA) regulates diverse processes of plant growth and development. It has recently been proposed that GCR2 functions as a G-protein-coupled receptor (GPCR) for ABA. However, the structural relationships and functionality of GCR2 have been challenged by several independent studies. A central question in this controversy is whether *gcr2* mutants are insensitive to ABA, because *gcr2* mutants were shown to display reduced sensitivity to ABA under one experimental condition (e.g. 22°C, continuous white light with 150 µmol m^-2^ s^−1^) but were shown to display wild-type sensitivity under another slightly different condition (e.g. 23°C, 14/10 hr photoperiod with 120 µmol m^−2^ s^−1^). It has been hypothesized that *gcr2* appears only weakly insensitive to ABA because two other *GCR2*-like genes in Arabidopsis, *GCL1* and *GCL2*, compensate for the loss of function of *GCR2*.

**Principal Findings:**

In order to test this hypothesis, we isolated a putative loss-of-function allele of *GCL2*, and then generated all possible combinations of mutations in each member of the *GCR2* gene family. We found that all double mutants, including *gcr2 gcl1*, *gcr2 gcl2*, *gcl1 gcl2*, as well as the *gcr2 gcl1 gcl2* triple mutant displayed wild-type sensitivity to ABA in seed germination and early seedling development assays, demonstrating that the *GCR2* gene family is not required for ABA responses in these processes.

**Conclusion:**

These results provide compelling genetic evidence that GCR2 is unlikely to act as a receptor for ABA in the context of either seed germination or early seedling development.

## Introduction

Abscisic acid (ABA) regulates diverse processes in plant growth and development, and is best known for its role in the inhibition of seed germination and early seedling development, and in the control of stomatal movement [1]. It has been demonstrated that the plant response to ABA requires some key signaling components, including protein kinases, protein phosphatases and transcription factors, and it has also been generally recognized that the ABA signal is perceived by multiple receptors, at the cell surface or inside the cells (recently reviewed in [2]–[5]). For example, FLOWERING TIME CONTROL PROTEIN A (FCA), a nuclear RNA-binding protein, has been proposed to be an ABA receptor controlling ABA-mediated RNA metabolism and flowering [6]. The H subunit of Mg-chelatase (CHLH), a chloroplast protein, has also been proposed to be an ABA receptor involved in mediating cellular responses to ABA during seed germination, post-germination growth and stomatal movement [7].

Recently, an additional receptor candidate, G-PROTEIN-COUPLED RECEPTOR 2 (GCR2), has been proposed to act as a plasma membrane receptor for ABA, controlling all the major responses mediated by ABA, including seed germination, early seedling development, stomatal movement and gene expression [8]. However, the structural relationships and functionality of GCR2 as an ABA-signaling G-protein-coupled receptor (GPCR) have been challenged by several independent studies [9]–[11]. While GCR2 was originally described as a classical GPCR [8], structural predictions from several robust transmembrane prediction systems subsequently showed that GCR2 was very unlikely to contain seven-transmembrane domains, a structural hallmark of any classical GPCRs. Instead, GCR2 was found to possess significant sequence and predicted structural similarity to homologs of bacterial lanthionine synthetase (LanC) in diverse species, suggesting that GCR2 may actually be a member of the LanC protein family [9], [10]. Recent Fourier transform re-analysis has provided an explanation why GCR2 was erroneously predicted to be a GPCR [11]. In light of these discrepancies, it has been proposed that GCR2 may define a new type of ‘non-classical’ GPCR required for ABA perception [12].

We had previously reported that *gcr2* mutants do not display consistent ABA insensitivity under our experimental conditions (e.g. 23°C, 14/10 hr photoperiod with 120 µmol m^−2^ s^−1^) [9]. However, since GCR2 is a member of small gene family, the possibility remained that *gcr2* mutants display little insensitivity to ABA because *GCR2* shares partial functional redundancy with two other *GCR2*-like genes in Arabidopsis, *GCR2-LIKE 1* (*GCL1*) and *GCR2-LIKE 2* (*GCL2*). In order to examine this possibility, we generated double and triple mutants between *gcr2*, *gcl1* and *gcl2*, and analyzed the sensitivity of these mutants to ABA in seed germination and early seedling development. Our results clearly demonstrate that none of the *GCR2*, *GCL1* or *GCL2* is required for ABA responses in seed germination or early seedling development, casting further doubt on the claim that GCR2 functions as an ABA receptor in these processes.

## Results

### *GCR2* homologs in Arabidopsis

**Figure 1:**
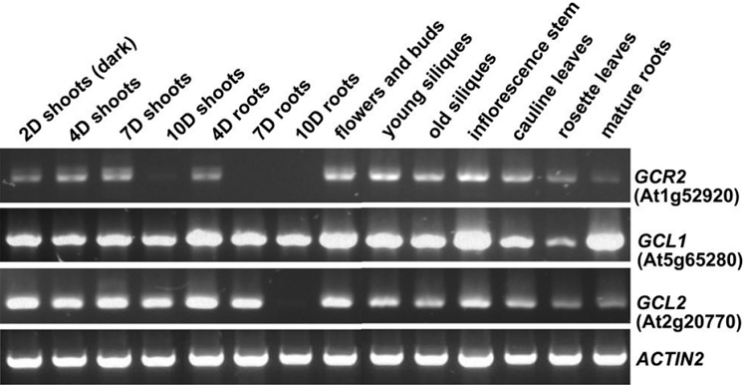
RT-PCR analysis of the expression of *GCR2*, *GCL1* and *GCL2* genes in various tissues and organs of young seedlings and mature plants. PCR was performed with 38 cycles. The expression of *ACTIN2* was used as a control.

The Arabidopsis genome contains two genes, *GCL1* (gene locus At5g65280) and *GCL2* (gene locus At2g20770), that encode proteins highly similar to GCR2 (Gao et al., 2007). Both *GCR2* and *GCL2* contain six exons and five introns with similar size distribution (Figure S1), while *GCL1* contains five exons and four introns. Similar to *GCR2*, *GCL1* and *GCL2* are expressed in dry seeds and the expression of all three genes decreases once seeds have germinated [9]. The similarities in amino acid sequence and in the expression profiles for these three genes suggest that *GCR2*, *GCL1* and *GCL2* may have overlapping function(s). When we conducted a more detailed expression analysis of the three genes across different tissues and organs in seedlings and adult plants, we found that their transcripts were present in various tissues/organs examined (Figure 1), and that some differences in expression pattern could be detected between *GCR2*, *GCL1* and *GCL2*. For example, transcripts of both *GCR2* and *GCL2* were undetectable in the roots of 10 d-old seedlings, and transcripts of *GCR2* were also undetectable in the roots of 7 d-old seedlings, whereas transcripts of *GCL1* were detected in all tissues/organs examined (Figure 1). These results indicate that, in addition to their activity in seeds, *GCR2*, *GCL1* and *GCL2* are also expressed in vegetative and floral tissues/organs, and that these three genes have both overlapping and non-overlapping expression patterns.

**Figure 2:**
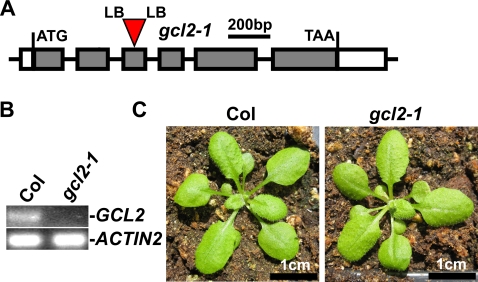
T-DNA insertion mutant of *gcl2*. (A) A diagram to illustrate the T-DNA insertion site in the *gcl2-1* mutant. Gray boxes represent exons and white boxes represent 5′- or 3′-UTR region. Introns are shown as lines between exons. The T-DNA insert is not drawn to scale. LB, T-DNA left border. (B) RT-PCR analysis of *GCL2* transcript in *gcl2-1* mutant. RNA was isolated from 7 d-old light-grown seedlings. PCR was performed with 40 cycles for *GCR2* and 30 cycles for *ACTIN2*. The expression of *ACTIN2* was used as a control. (C) *gcl2-1* mutant plants have wild-type morphology. Shown are 21 d-old plants grown under 14/10 hr photoperiod.

**Figure 3:**
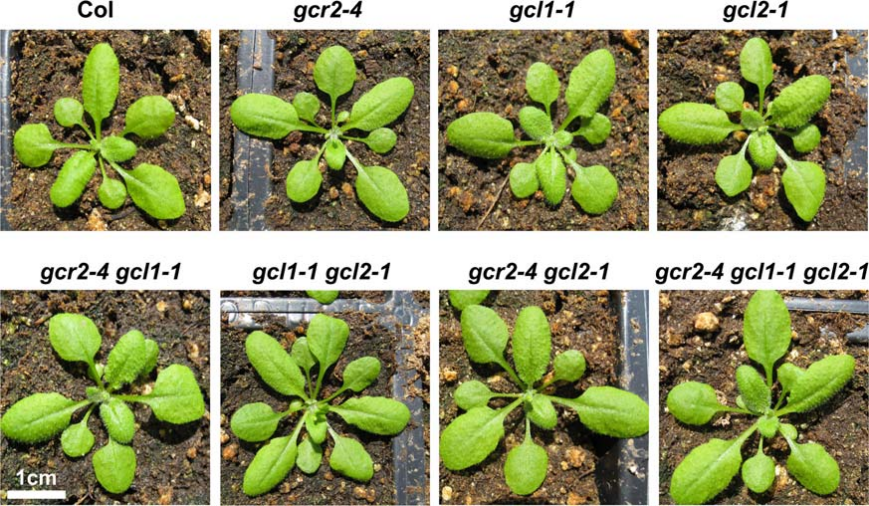
*gcr2*, *gcl1* and *gcl2* single, double and triple mutants. Shown are 21 d-old plants grown under 14/10 hr photoperiod.

**Figure 4:**
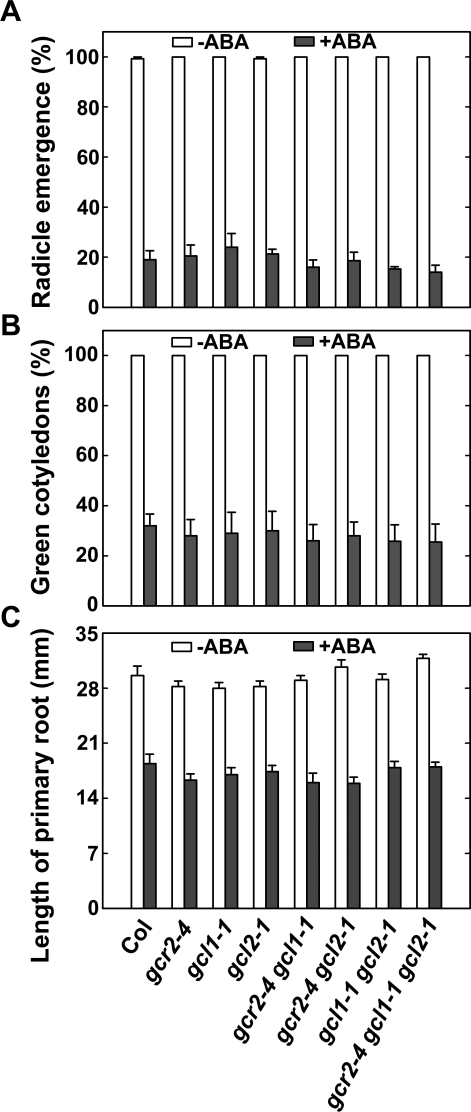
Sensitivities of *gcr2*, *gcl1* and *gcl2* single, double and triple mutants to ABA in seed germination and early seedling development. (A) ABA sensitivity of *gcr2*, *gcl1* and *gcl2* single, double and triple mutants in seed germination assay. ABA was used at 1.0 µM. The percentage of seeds with emerged radicles was scored 60 hr after the imbibed seeds were transferred to germination conditions. Shown are the averages of three replicates±S.E. (B) ABA sensitivity of *gcr2*, *gcl1* and *gcl2* single, double and triple mutants in the green seedling assay. ABA was used at 1.0 µM. The percentage of seedlings with green cotyledons was scored 11 d after the imbibed seeds were transferred to germination conditions. Shown are the averages of three replicates±S.E. (C) ABA sensitivity of *gcr2*, *gcl1* and *gcl2* single, double and triple mutants in the primary root elongation inhibition assay. ABA was used at 2.0 µM. Four-eight hours after the imbibed seeds were transferred to germination conditions, germinated seeds with emerged radicles were then transferred to medium with or without ABA. The length of primary root was measured 5 d later. Shown are the averages of at least 10 seedlings±S.E.

### Mutant allele of *GCL2*

Within the *GCR2* gene family, mutant alleles for only *GCR2* and *GCL1* have been reported previously [8], [9]. We report here the isolation and characterization of a *gcl2* mutant allele. By searching the SALK Institute sequence-indexed insertion mutant collection (http://signal.salk.edu/cgi-bin/tdnaexpress) and the GABI-KAT database (http://www.gabi-kat.de/), we identified one T-DNA insertion mutant of the *GCL2* gene, GABI_115D02. This allele, which possesses a T-DNA insertion within the 3^rd^ exon of the *GCL2* gene, was designated as *gcl2-1* (Figure 2A, Figure S2). RT-PCR analysis indicated that full-length *GCL2* transcript is absent in *gcl2-1*, implying that this is likely a loss-of-function allele of *GCL2* (Figure 2B). The *gcl2-1* mutant does not display any obvious morphological phenotypes (Figure 2C).

### Double and triple mutants between *gcr2*, *gcl1* and *gcl2*

Because putative loss-of-function mutant alleles are now available for each member of *GCR2* gene family, we generated all possible double and triple mutants between *gcr2*, *gcl1* and *gcl2* in an effort to examine possible functional redundancy of these genes. We have previously reported the characterization of *gcr2 gcl1* double mutants [9], and here we report the other double mutants, including *gcr2 gcl2* and *gcl1 gcl2*, as well as the *gcr2 gcl1 gcl2* triple mutant. We found that neither the double mutants nor the triple mutant displayed any obvious morphological phenotypes under normal growth conditions (e.g. 14/10 hr photoperiod with approximately 120 µmol m^−2^ s^−1^ at 23°C) (Figure 3).

Subsequently, we used the simple, robust ABA-inhibition of seed germination and early seedling development assays to examine the ABA sensitivity of these mutants. First, we scored the percentage of seeds displaying radicle emergence in the presence of ABA as an accepted metric for the impact of these mutations on the ABA response during seed germination. We found that all single, double and triple mutants behaved like wild-type in this assay (Figure 4A). Next, we scored the percentage of green seedlings (seedlings with green cotyledons) when grown in the presence of ABA, as a measure of the impact of these mutations on the ABA response of seedlings during early development. As in the previous assay, we found that all single, double and triple mutants behaved like wild-type (Figure 4B). Finally, we used the ABA-inhibition of primary root growth assay to establish whether these mutants display insensitivity to ABA. In this assay, stratified seeds were imbibed on MS medium without ABA for 48 hr under normal germination conditions (23°C, 14/10 hr photoperiod at 120 µmol m^−2^ s^−1^). Then, germinated seeds with emerged radicles were transferred to MS medium containing ABA. This assay allows us to specifically examine post-germination ABA sensitivity of these mutants. We found that all single, double and triple mutants again behaved like wild-type in this assay (Figure 4C).

## Discussion

GCR2 has been proposed to represent a plasma membrane receptor for ABA mediating all major ABA responses [8], [12], but studies from our laboratory and from others have challenged this model [9], [10]. In order to bring more evidence to bear on the controversy surrounding this topic, in particular, on the question of possible functional redundancy of *GCR2* and *GCR2*-like genes in ABA signalling, we report here the examination of the impact of all possible combinations of mutations in each member of *GCR2* gene family on the ABA control of seed germination and early seedling development. Our results indicate that GCR2 is unlikely to be a functional ABA receptor mediating ABA responses in seed germination and early seedling development.

### The *GCR2* gene family is not required for ABA responses in seed germination and early seedling development

*GCR2* belongs to a small gene family in Arabidopsis that consists of *GCR2* and two additional genes, *GCL1* and *GCL2*. The latter two genes encode proteins that are highly similar to GCR2 [9]. Because the *gcr2* mutant displayed only slightly reduced sensitivity to ABA in seed germination and early seedling development assays under one experimental condition (e.g. 22°C, continuous white light with 150 µmol m^−2^ s^−1^) [8] while behaving like wild-type under another slightly different condition (e.g. 23°C, 14/10 hr photoperiod with 120 µmol m^−2^ s^−1^) [9], the ABA sensitivity of *gcr2* mutants has been controversial. One hypothesis was that *gcr2* mutants could have weak ABA phenotypes because *GCR2* functions redundantly with *GCL1* and *GCL2* in ABA signalling. Similar scenarios have been documented for other plant hormone receptors. For example, although TIR1 functions as an auxin receptor [13], [14], *tir1* single mutants display weak auxin phenotypes, whereas severe auxin phenotypes are observed in double, triple and quadruple mutants of the *TIR1* gene family members [15]. Therefore, if GCR2 indeed functions as an ABA receptor and is functionally redundant with other members of the *GCR2* gene family, one would expect to observe an enhanced ABA insensitivity in the corresponding double and triple mutants. To test this hypothesis, we generated all possible combinations of mutations in each member of the *GCR2* gene family. By using the accepted and robust ABA-mediated inhibition of seed germination and early seedling development assays, we demonstrated that none of the *gcr2*, *gcl1* and *gcl2* single, double and triple mutants displayed ABA insensitivity (Figure 4). These results support our previous findings using *gcr2* and *gcl1* single and double mutants [9], and provide further evidence that the *GCR2* gene family is not required for ABA responses in seed germination and early seedling development.

### What is the function of the *GCR2* gene family?

GCR2, GCL1 and GCL2 are all highly similar at the amino acid level to LanC-like (LANCL) proteins in mammals, and they possess all the structural features that categorize them as members of the LANCL protein family [16]. Prokaryotic LanC proteins are involved in the modification and transport of peptides by acting in concert with specific dehydratases to facilitate intramolecular conjugation of cysteine to serine or threonine residues, yielding macrocyclic thioether (lanthionine) products. These products display antimicrobial activity and are known as lantibiotics (reviewed in [17], [18]). However, the presence of macrocyclic lanthionine-containing polypeptides similar to the prokaryotic lantibiotic structure has not yet been documented in higher eukaryotes. Therefore, the exact biological role of LANCL proteins is unclear. The *gcr2*, *gcl1* and *gcl2* single, double and triple mutants do not display any apparent morphological phenotypes (Figure 2, Figure 3), offering little clue to the exact biological role of *GCR2* gene family in Arabidopsis. This lack of a marked deficiency phenotype implies that the *GCR2* gene family may not be critical for plant development, but leaves open the possibility that these mutants may only exhibit phenotypes under certain conditions (e.g. under biotic and abiotic stresses).

We took several approaches to investigate the possible functions of the *GCR2* gene family. Because GCR2 has been shown to physically bind to GPA1 [8] and GCL1 and GCL2 are highly similar to GCR2 at the amino acid level [9], we first examined a possible role for these proteins in G-protein signaling. First, we examined the possibility of GCL1 and GCL2 being classical GPCRs. Structural analysis *in silico* using the TMHMM2.0 (http://www.cbs.dtu.dk/services/TMHMM/) transmembrane segment prediction system predicted no transmembrane domains in GCL1 and GCL2 (Figure S3), similar to the situation for GCR2 [9]. This implies that the GCR2 protein family members unlikely function as classical GPCRs, because they do not possess seven-transmembrane domains, a structural hallmark of any classical GPCRs. Second, because Liu et al. (2007) recently argued that GCR2 may define a new type of non-classical GPCR [12], we sought other evidence that may support a genetic coupling between the GCR2 protein family and G-proteins. The G-protein repertoire is much simpler in plants (reviewed in [19]–[22]), and Arabidopsis possesses only one canonical G-protein α subunit, one G-protein β subunit, and two G-protein γ subunit. We reasoned that, if GCR2 family proteins are important contributors to G-protein-coupled signalling pathways, one would expect to observe shared phenotypes (morphological or conditional) between G-protein subunit mutants and *GCR2*/*GCL* mutants. One of the characteristic morphological traits of loss-of-function alleles of the sole Arabidopsis heterotrimeric G-protein α subunit, *GPA1*, and the sole Arabidopsis heterotrimeric G-protein β subunit, *AGB1*, is round shaped rosette leaves [23]–[25], but none of the *gcr2*, *gcl1* and *gcl2* single, double and triple mutants displayed such morphological defects (Figure 3). Another characteristic trait of *gpa1* and *agb1* mutants is the short hypocotyl and partially-opened hook phenotype in etiolated seedlings [23]–[25]. Again, none of the *gcr2*, *gcl1* and *gcl2* single, double and triple mutants displayed such phenotypes (Figure S4). Therefore, although GCR2 has been shown to physically interact with GPA1 [8], the exact role of GCR2 (and GCL1 and GCL2) in G-protein signaling remains unclear.

We explored indirectly the possibility that *gcr2*, *gcl1* and *gcl2* might possess conditional phenotypes by examining the stress-specific expression profile of *GCR2*, *GCL1* and *GCL2* genes in the Genevestigator *Arabidopsis thaliana* microarray database (https://www.genevestigator.ethz.ch/) [26]. We found that the expression of *GCR2*, *GCL1* and *GCL2* can be up- or down-regulated by certain stresses, but in general, the fold-change in transcript abundance was modest (Figure S5). Very few treatments can up- or down-regulate the transcription of *GCR2*, *GCL1* or *GCL2* more than two-fold. This indirect approach provided little information about specific biotic or abiotic conditions that would warrant testing the behaviour of *gcr2* mutants directly.

Finally, we directly examined the sensitivity of *gcr2* mutants to other plant hormones including auxin, cytokinin, gibberellin, ethylene and brassinosteroid, because these hormones regulate almost every aspect of plant growth and development. We used the root elongation inhibition assay for measuring the sensitivity of *gcr2* mutants to auxin, cytokinin and brassinosteroid, used the hypocotyl elongation promotion assay for measuring the sensitivity to gibberellin, and used the triple response assay for measuring the sensitivity to ethylene. All *gcr2*, *gcl1* and *gcl2* single, double and triple mutants behaved like wild-type in all of these assays (Figure S6, Figure S7 and Figure S8).

In summary, our results from the examination of mutants carrying all possible combinations of mutations in each member of *GCR2* gene family suggest that GCR2 is not required for the ABA control of seed germination and early seedling development, and do not support the hypothesis that GCR2 functions as a receptor of ABA in these processes. Because we have yet discovered any morphological or conditional phenotypes in *gcr2* mutants, the exact role of *GCR2* in plants remains unknown.

## Materials and Methods

### Plant materials and growth conditions

All mutants are in the Arabidopsis Columbia ecotypic background (Col-0). *gcr2-4* and *gcl1-1* single mutants have been described previously [9]. Plants were grown in 4 × 4 cm pots containing moistened Sunshine Mix #4 (Sun Gro Horticulture Canada Ltd., www.sungro.com) under 14/10 hr photoperiod with approximately 120 µmol m^−2^ s^−1^ at 23°C, unless specified elsewhere.

### Isolation of T-DNA insertion mutant of *GCL2*

A T-DNA insertion mutant allele of *GCL2* (At2g20770), GABI_115D02, was identified from the GABI-KAT (http://www.gabi-kat.de/) database. Mutants homozygous for the insertion locus were isolated by PCR genotyping using *GCL2*-specific primers (5′-ATGGCGGGTCGGTTCTTCGATAAC-3′ and 5′-GGCTTCAGCTGAACATCCATC-3′) and a T-DNA left border-specific primer (5′-ATATTGACCATCATACTCATTGC-3′). The insertion site was confirmed by sequencing. The T-DNA was found to insert in the 3^rd^ exon of the *GCL2* gene. This mutant allele was designated as *gcl2-1*.

### Generation of *gcr2*, *gcl1* and *gcl2* double and triple mutants

The *gcr2-4 gcl1-1* double mutants have been described previously (Gao et al., 2007). The *gc1-1 gcl2-1* double mutant was generated by crossing *gcl2-1* with *gcl1-1* and isolated in the F2 progeny by PCR genotyping. The *gcr2-4 gcl2-1* double mutant and *gcr2-4 gcl1-1 gcl2-1* triple mutant were generated by crossing *gcl2-1* single mutant with *gcr2-4 gcl1-1* double mutant and isolated in the F2 progeny by PCR genotyping. All double and triple mutants were confirmed by subsequent RT-PCR analyses. For simplicity, the *gcr2 gcl1, gcr2 gcl2* and *gcl1 gcl2* nomenclatures in this report refer specifically to the *gcr2-4 gcl1-1*, *gcr2-4 gcl2-1* and *gcl1-1 gcl2-1* double mutants, respectively. The *gcr2 gcl1 gcl2* nomenclature refers specifically to the *gcr2-4 gcl1-1 gcl2-1* triple mutant.

### ABA inhibition of seed germination and early seedling development assays

Wild-type and mutant seeds from matched lots were surface-sterilized with 80% ethanol for 2 min, followed by 50% bleach with 0.1% Tween-20 for 10 min, and washed with sterile, de-ionized water five times. Sterilized seeds were then directly sown on MS/G plates consisting of ½ Murashige & Skoog (MS) basal medium supplemented with vitamins (Plantmedia, www.plantdemia.com), 1% (w/v) sucrose, 0.6% (w/v) phytoagar (Plantmedia), pH adjusted to 5.7 with 1N KOH, and with or without ABA. Imbibed seeds were cold-treated at 4°C in dark for 2 days, and then moved to 23°C, with 14/10 hr photoperiod (120 µmol m^−2^ s^−1^). The percentage of seed germination was scored every 12 hr, and was examined under a dissecting microscope. Germination is defined as an obvious protrusion of the radicle through the seed coat. Each experiment was conducted with three replicates.

For the ABA inhibition of early seedling development, two different assays were used. In the first assay, the percentage of seedlings with green cotyledons was scored from seeds germinated on MS/G plates containing 1.0 µM ABA for 11 days. In the second assay, the ABA inhibition of primary root growth was measured. Surface-sterilized wild-type and mutant seeds were imbibed on MS/G medium and cold-treated at 4°C in dark for 2 days. Then, imbibed seeds were placed under germination conditions (23°C, 14/10 hr photoperiod at 120 µmol m^−2^ s^−1^). Four-eight hours later, germinated seeds with emerged radicle were transferred to MS/G medium containing 2.0 µM ABA. The length of primary root was monitored every 24 hr. At least 10 seedlings were used for each genotype.

### RT-PCR

RT-PCR was used to analyze the expression of *GCR2*, *GCL1* and *GCL2* in various tissues/organs. Total RNA was isolated from different parts of seedlings or mature plants using the TRIzol reagent (Invitrogen, www.invitrogen.com). cDNA was synthesized from 1 µg total RNA by oligo(dT)_20_-primed reverse transcription using THERMOSCRIPT RT (Invitrogen). *GCR2*-specific primers (5′-GGAAGATTTATCCGGAGAAGAAGAAACTGT-3′, and 5′-CAGAGCTTGTGTTGGATCATTCATGTCG-3′), *GCL1*-specific primers (5′-ATGTCGTCGTCGGTGGAT-3′ and 5′-TTATATCTCATAACCAGGA-3′), and *GCL2*-specific primers (5′-ATCCGCGAAGTTGCTCAGGA-3′ and 5′-TTAAAGCTCGTAACCTGGGAGAAG-3′) were used to amplify the transcript of these three genes. *ACTIN2* (amplified by primers 5′-CCAGAAGGATGCATATGTTGGTGA-3′ and 5′-GAGGAGCCTCGGTAAGAAGA-3′) was used as a control in PCR reactions.

## Supporting Information

Figure S1 The gene structure of GCR2, GCL1 and GCL2. The genomic DNA sizes for GCR2, GCL1 and GCL2 are 1778 bp, 2181 bp and 1993 bp, respectively. The full-length CDs for GCR2, GCL1 and GCL2 are 1233 bp, 1302 bp and 1218 bp, respectively. The proteins encoded by GCR2, GCL1 and GCL2 are 410 aa, 433 aa and 405 aa long, respectively. Both GCR2 and GCL2 contain six exons and five introns with similar sizes. GCL1 contains five exons and four introns. Gray box, exon; white box, 5′- or 3′-UTR region. Introns are shown as lines between exons.
(0.13 MB TIF)

Figure S2 T-DNA insertion site in gcl2-1 mutant. Genomic DNA from mutant plants homozygous for the gcr2-1 locus was used to amplify DNA fragments in PCR reactions. GCL2 gene-specific forward or reverse primers and the T-DNA left border (LB) primer were used in PCR reactions. The T-DNA insertion site was confirmed by sequencing. In gcr2-1 allele, a tandem T-DNA with two outward facing LB was inserted in the 3rd exon of the GCL2 gene. In this allele, the T-DNA insertion resulted in the deletion of 48 bp from the 3rd exon of GCL2 gene. Lower case letters indicate 5′- and 3′-UTR regions or introns. The positions of T-DNA left border (LB) are shown.
(2.53 MB TIF)

Figure S3 Transmembrane domain predictions for GCL1 and GCL2. The transmembrane segment prediction for GCL1 (A) and GCL2 (B) was performed using the TMHMM2.0 (http://www.cbs.dtu.dk/services/TMHMM/).
(0.90 MB TIF)

Figure S4 Etiolated seedlings of gcr2, gcl1 and gcl2 single, double and triple mutants. Shown are 2.5 d-old seedlings grown under darkness.
(2.99 MB TIF)

Figure S5 The in silico analysis of the expression of GCR2 (At1g52920), GCL1 (At5g65280), and GCL2 (At2g20770) in response to various treatments. Data were imported from Genevestigator Arabidopsis thaliana microarray database (https://www.genevestigator.ethz.ch/). Only treatments that induce > = 2.0-fold change in the transcript level of GCR2, GCL1 or GCL2 are shown. Number of chips used in each treatment is indicated in parentheses. Ratios of treatment to non-treatment control are scaled with different colors.
(1.08 MB TIF)

Figure S6 Sensitivities of gcr2, gcl1 and gcl2 single, double and triple mutants to auxin, cytokinin and brassinosteroid in the root elongation inhibition assays. Wild-type and mutant seeds were germinated on MS/G medium without hormones. Three days later, seedlings were transferred to Petri-dishes containing MS/G medium and individual hormone, and the Petri-dishes were placed vertically to monitor primary root growth. The length of primary root was measured five days later. Shown are the averages of at least 10 seedlings±S.E. (A) Sensitivities to auxin. Synthetic auxin, 1-naphthaleneacetic acid (NAA), was used at 0.5 µM. (B) Sensitivities to cytokinin. Synthetic cytokinin, 6-benzylaminopurine (6-BA), was used at 1.0 µM. (C) Sensitivities to brassinosteroid. 24-epibassinolide (24-epiBL) was used at 0.1 µM.
(1.05 MB TIF)

Figure S7 Sensitivities of gcr2, gcl1 and gcl2 single, double and triple mutants to gibberellin in the hypocotyl elongation assay. Surface-sterilized wild-type and mutant seeds were directly sown on MS/G medium with or without 10 µM GA3 and cold-treated at 4°C in dark for 2 days. Then, imbibed seeds were transferred to growth conditions (23°C, 14/10 hr photoperiod at 120 µmol m-2 s-1). Four days later, the length of hypocotyl was measured. Shown are the averages of at least 15 seedlings±S.E.
(0.22 MB TIF)

Figure S8 Sensitivities of gcr2, gcl1 and gcl2 single, double and triple mutants to ethylene in the triple response assay. Surface-sterilized wild-type and mutant seeds were directly sown on MS/G medium with or without 10 µM 1-aminocyclopropane-1-carboxylic acid (ACC), an ethylene precursor, and cold-treated at 4°C in dark for 2 days. Then, imbibed seeds were transferred to 23°C in dark. Shown are 3 d-old, dark-grown seedlings in the presence of 10 µM ACC. (A) The phenotype of gcr2, gcl1 and gcl2 single, double and triple mutants in response to 10 µM ACC. (B) The hook region of wild-type Col (left) and gcr2 gcl1 gcl2 triple mutant (right) in the presence of 10 µM ACC.
(5.30 MB TIF)
