# Supplementary figures and images for: The GCR2 Gene Family Is Not Required for ABA Control of Seed Germination and Early Seedling Development in Arabidopsis
Source: PLoS One. 2008 Aug 20;3(8):e2982. doi: 10.1371/journal.pone.0002982 (PMC2500181; doi:10.1371/journal.pone.0002982)

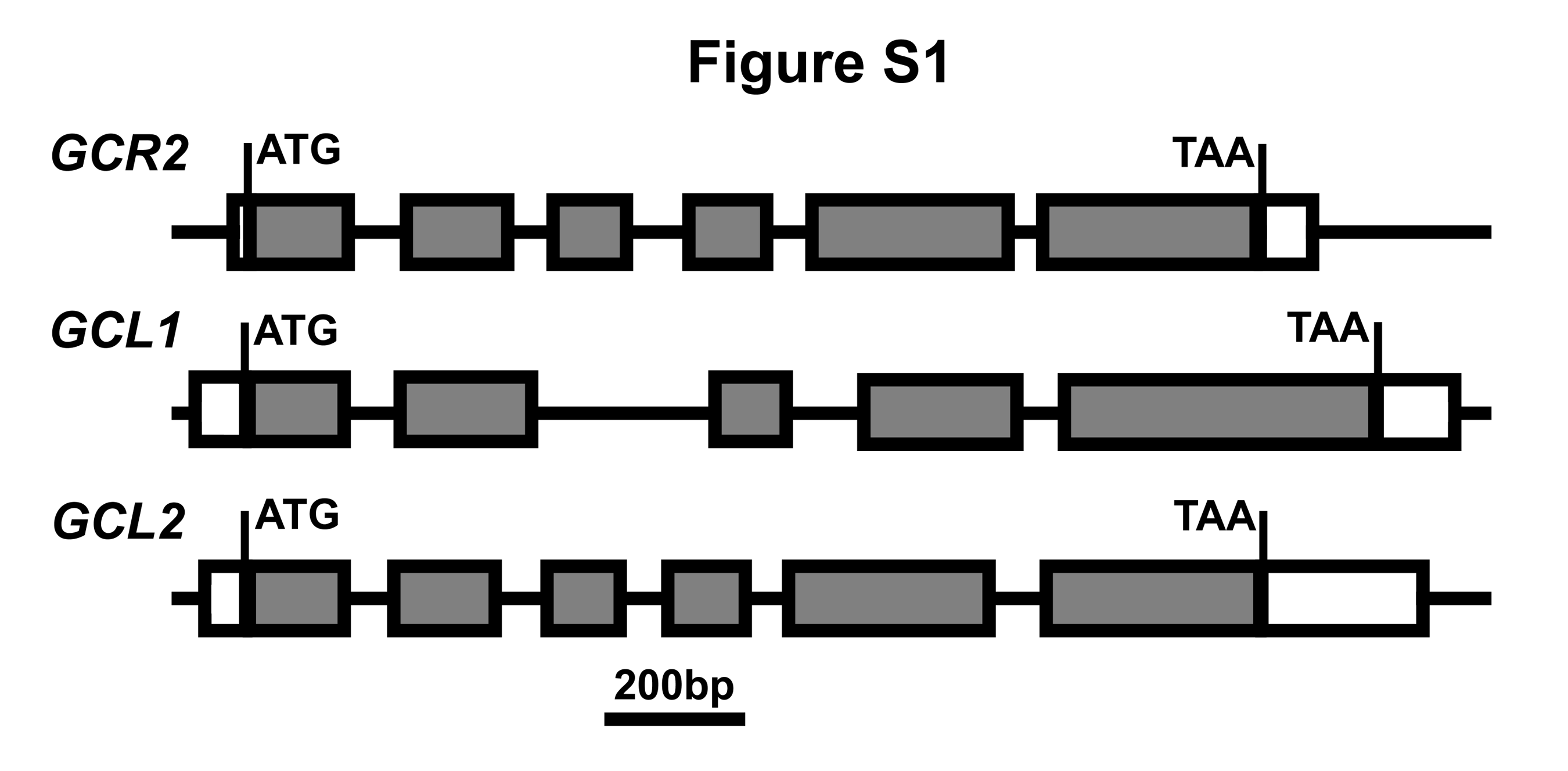

Supplement: Figure S1 — The gene structure of GCR2, GCL1 and GCL2. The genomic DNA sizes for GCR2, GCL1 and GCL2 are 1778 bp, 2181 bp and 1993 bp, respectively. The full-length CDs for GCR2, GCL1 and GCL2 are 1233 bp, 1302 bp and 1218 bp, respectively. The proteins encoded by GCR2, GCL1 and GCL2 are 410 aa, 433 aa and 405 aa long, respectively. Both GCR2 and GCL2 contain six exons and five introns with similar sizes. GCL1 contains five exons and four introns. Gray box, exon; white box, 5′- or 3′-UTR region. Introns are shown as lines between exons. (0.13 MB TIF) [file pone.0002982.s001.tif]

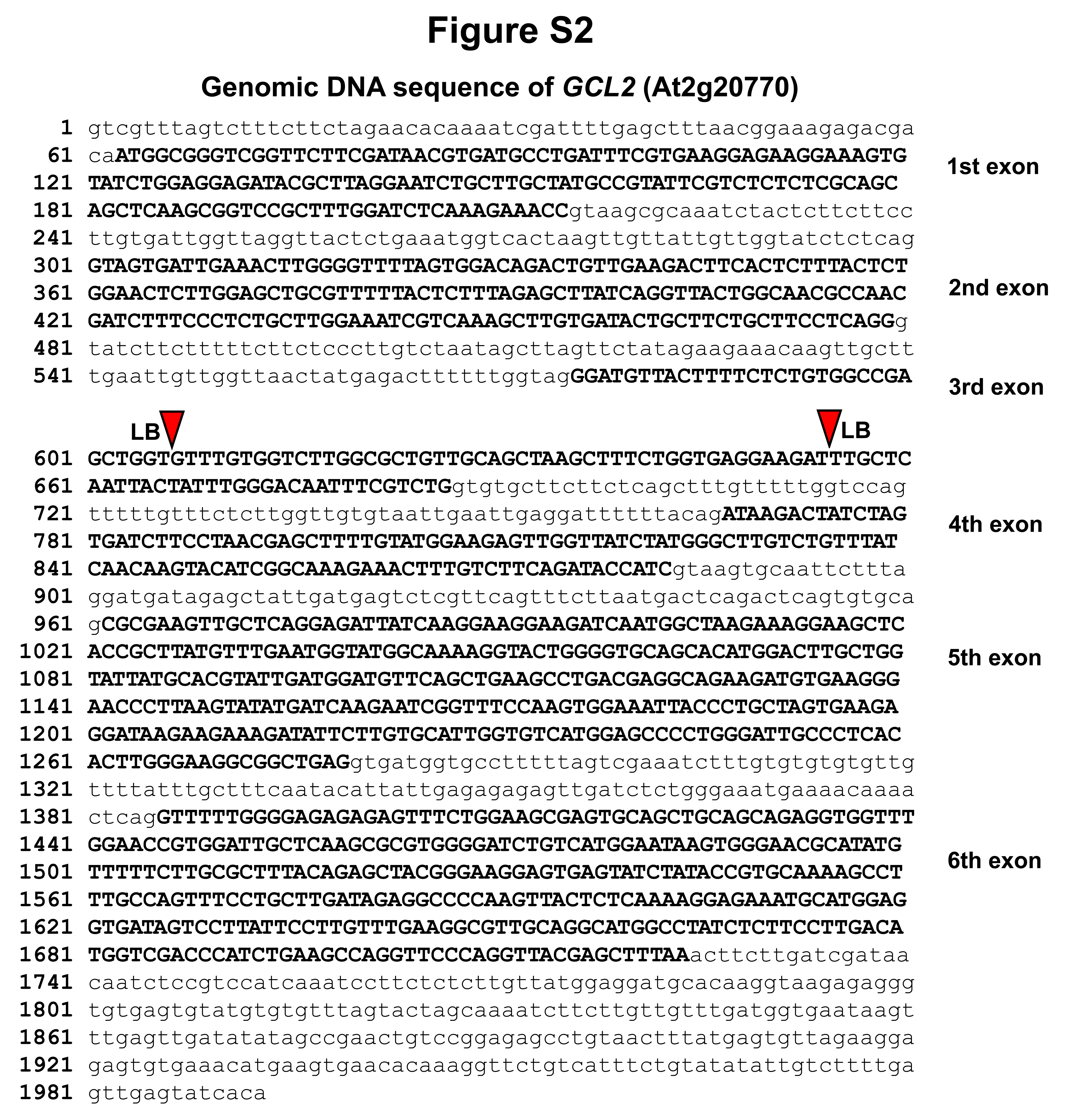

Supplement: Figure S2 — T-DNA insertion site in gcl2-1 mutant. Genomic DNA from mutant plants homozygous for the gcr2-1 locus was used to amplify DNA fragments in PCR reactions. GCL2 gene-specific forward or reverse primers and the T-DNA left border (LB) primer were used in PCR reactions. The T-DNA insertion site was confirmed by sequencing. In gcr2-1 allele, a tandem T-DNA with two outward facing LB was inserted in the 3rd exon of the GCL2 gene. In this allele, the T-DNA insertion resulted in the deletion of 48 bp from the 3rd exon of GCL2 gene. Lower case letters indicate 5′- and 3′-UTR regions or introns. The positions of T-DNA left border (LB) are shown. (2.53 MB TIF) [file pone.0002982.s002.tif]

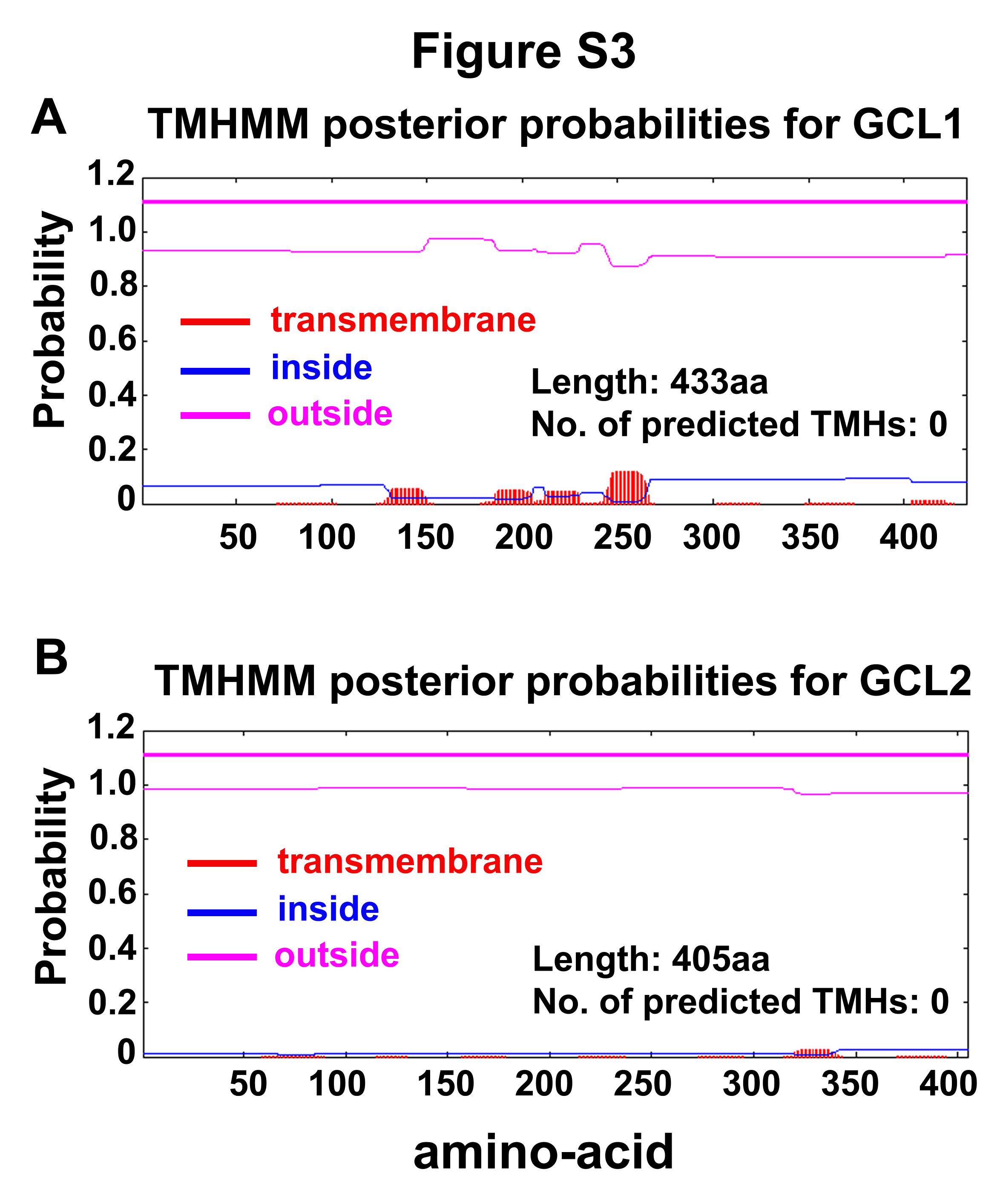

Supplement: Figure S3 — Transmembrane domain predictions for GCL1 and GCL2. The transmembrane segment prediction for GCL1 (A) and GCL2 (B) was performed using the TMHMM2.0 (http://www.cbs.dtu.dk/services/TMHMM/). (0.90 MB TIF) [file pone.0002982.s003.tif]

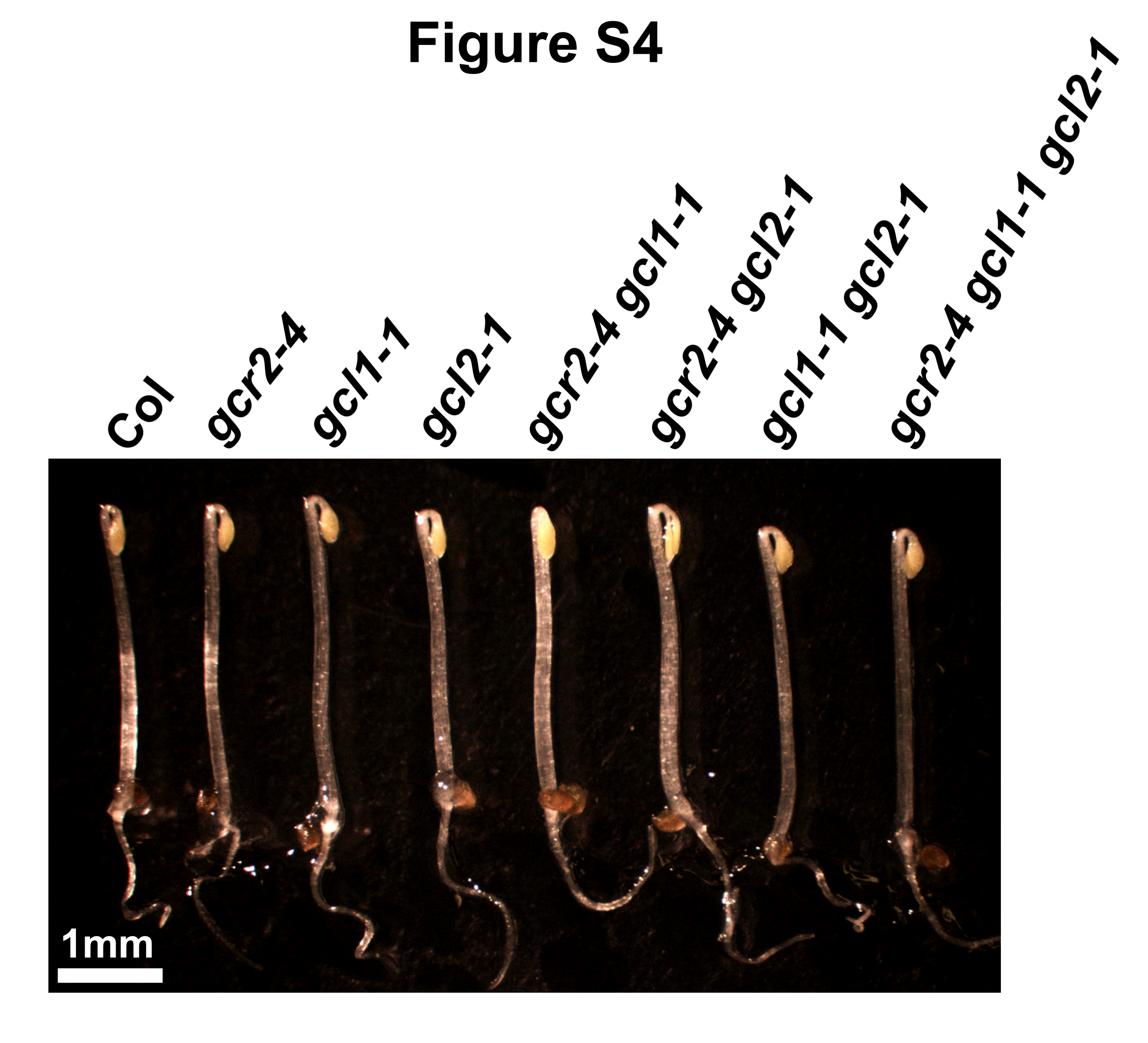

Supplement: Figure S4 — Etiolated seedlings of gcr2, gcl1 and gcl2 single, double and triple mutants. Shown are 2.5 d-old seedlings grown under darkness. (2.99 MB TIF) [file pone.0002982.s004.tif]

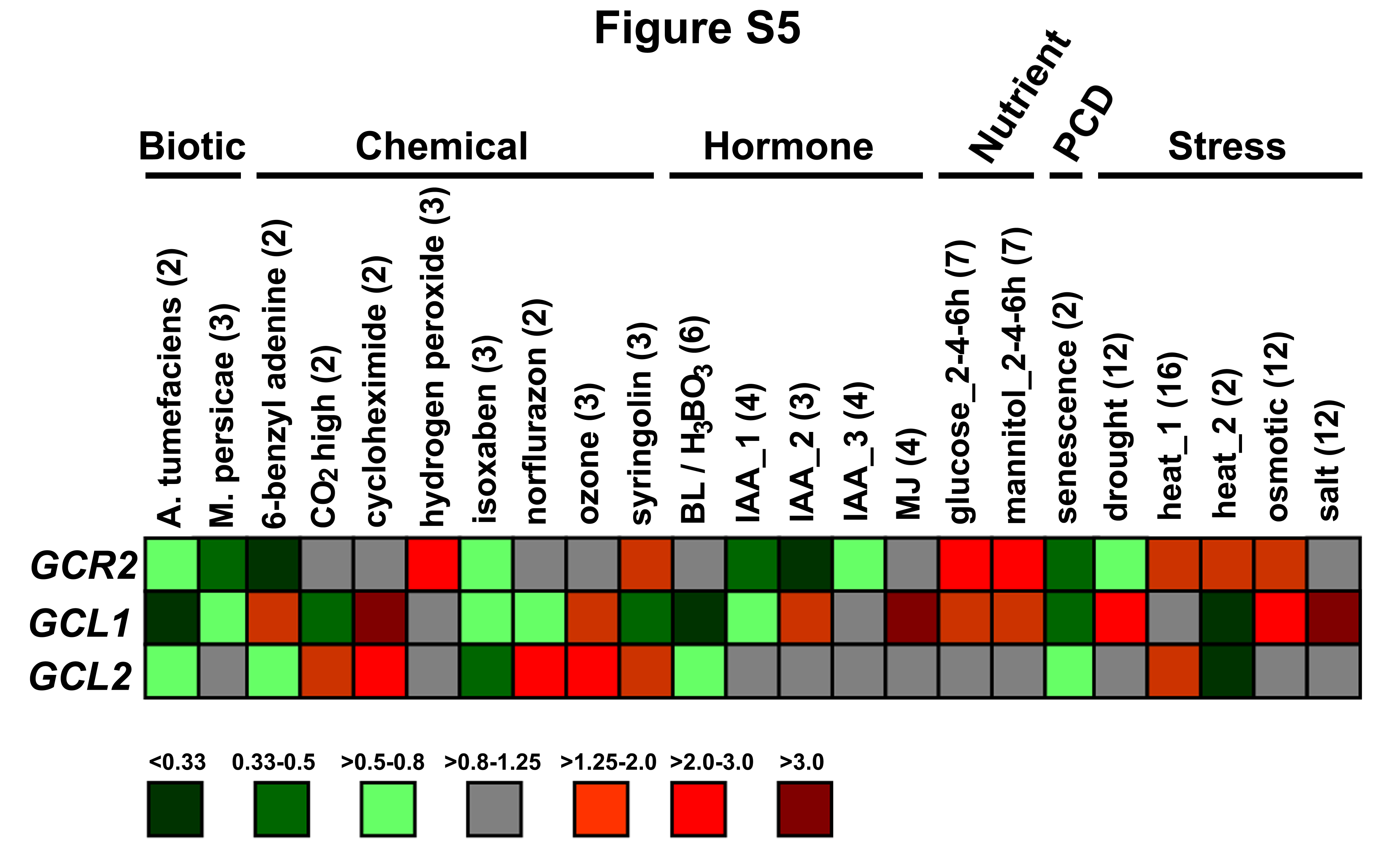

Supplement: Figure S5 — The in silico analysis of the expression of GCR2 (At1g52920), GCL1 (At5g65280), and GCL2 (At2g20770) in response to various treatments. Data were imported from Genevestigator Arabidopsis thaliana microarray database (https://www.genevestigator.ethz.ch/). Only treatments that induce > = 2.0-fold change in the transcript level of GCR2, GCL1 or GCL2 are shown. Number of chips used in each treatment is indicated in parentheses. Ratios of treatment to non-treatment control are scaled with different colors. (1.08 MB TIF) [file pone.0002982.s005.tif]

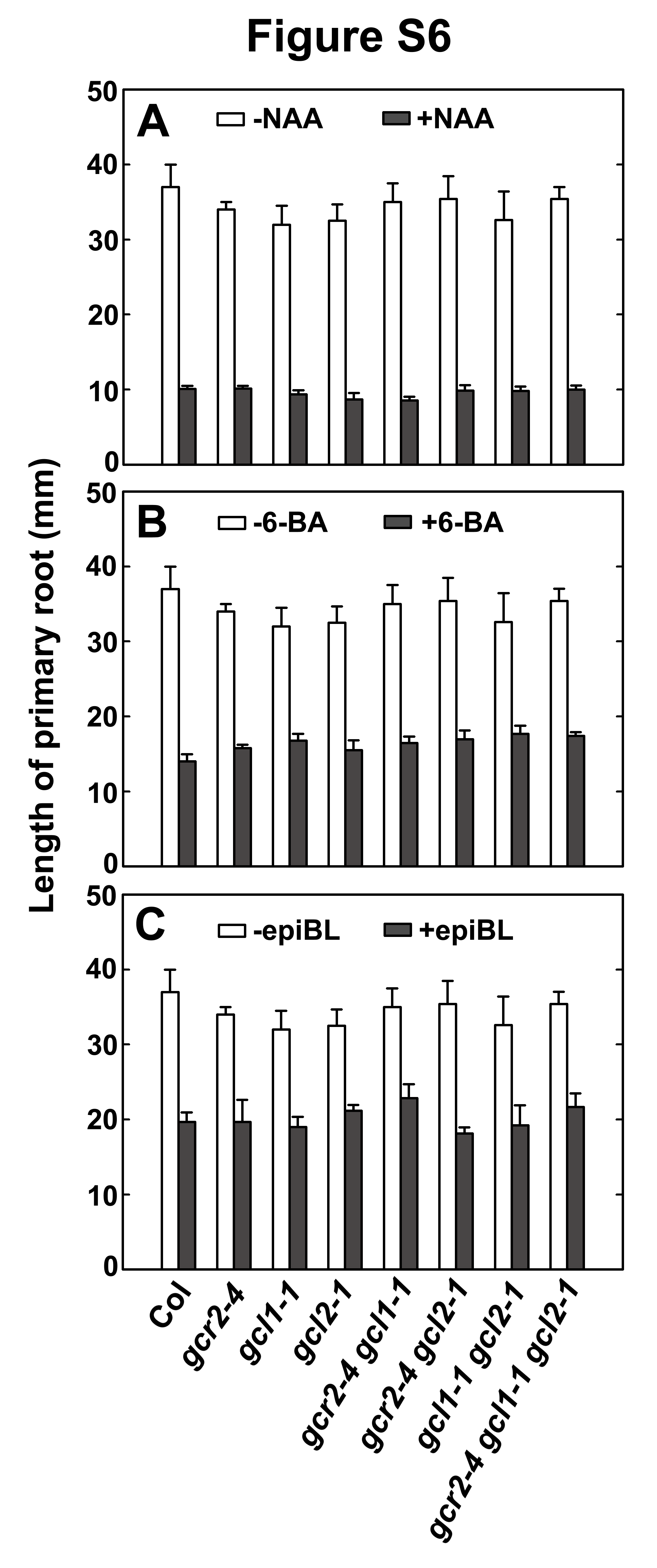

Supplement: Figure S6 — Sensitivities of gcr2, gcl1 and gcl2 single, double and triple mutants to auxin, cytokinin and brassinosteroid in the root elongation inhibition assays. Wild-type and mutant seeds were germinated on MS/G medium without hormones. Three days later, seedlings were transferred to Petri-dishes containing MS/G medium and individual hormone, and the Petri-dishes were placed vertically to monitor primary root growth. The length of primary root was measured five days later. Shown are the averages of at least 10 seedlings±S.E. (A) Sensitivities to auxin. Synthetic auxin, 1-naphthaleneacetic acid (NAA), was used at 0.5 µM. (B) Sensitivities to cytokinin. Synthetic cytokinin, 6-benzylaminopurine (6-BA), was used at 1.0 µM. (C) Sensitivities to brassinosteroid. 24-epibassinolide (24-epiBL) was used at 0.1 µM. (1.05 MB TIF) [file pone.0002982.s006.tif]

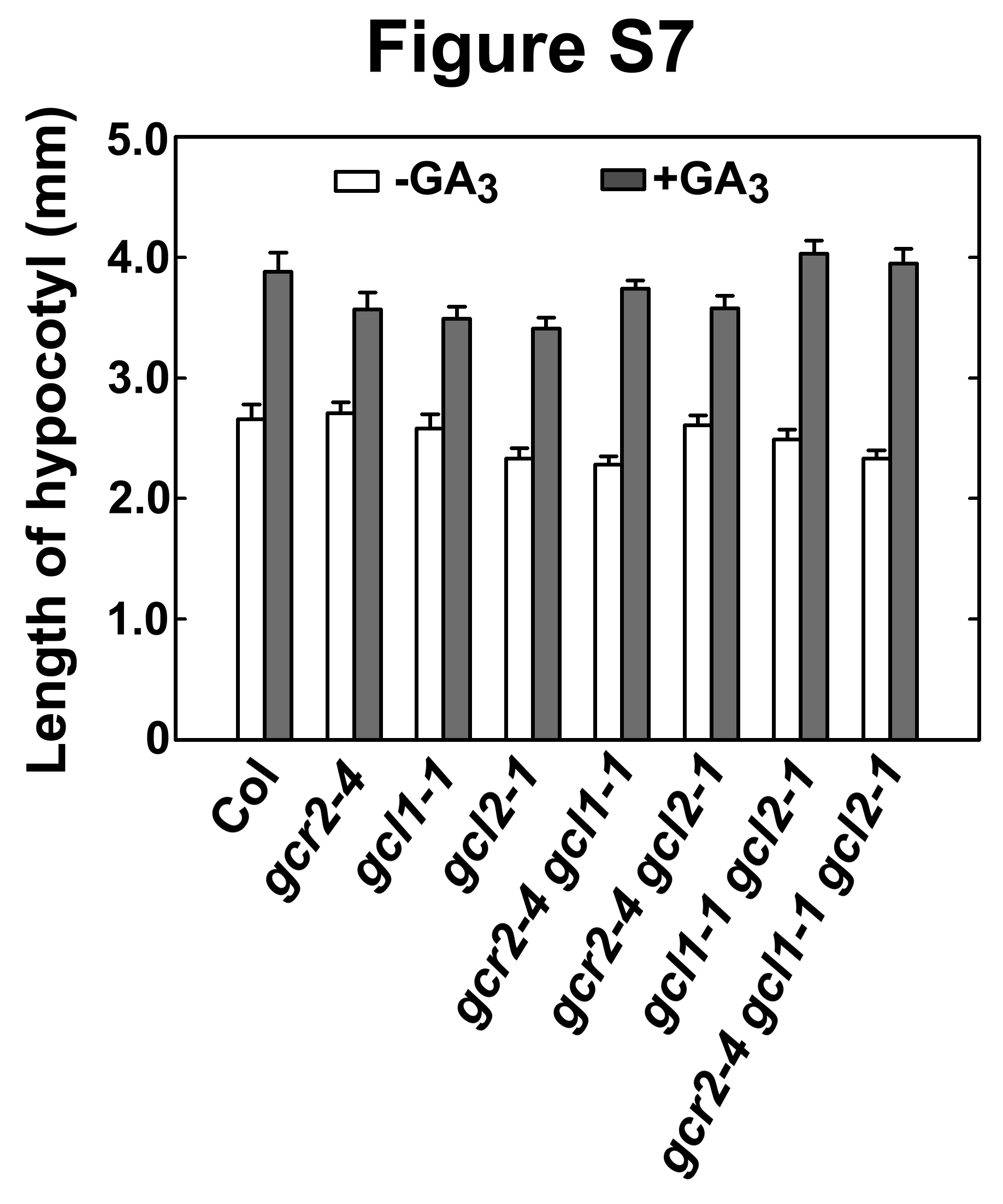

Supplement: Figure S7 — Sensitivities of gcr2, gcl1 and gcl2 single, double and triple mutants to gibberellin in the hypocotyl elongation assay. Surface-sterilized wild-type and mutant seeds were directly sown on MS/G medium with or without 10 µM GA3 and cold-treated at 4°C in dark for 2 days. Then, imbibed seeds were transferred to growth conditions (23°C, 14/10 hr photoperiod at 120 µmol m-2 s-1). Four days later, the length of hypocotyl was measured. Shown are the averages of at least 15 seedlings±S.E. (0.22 MB TIF) [file pone.0002982.s007.tif]

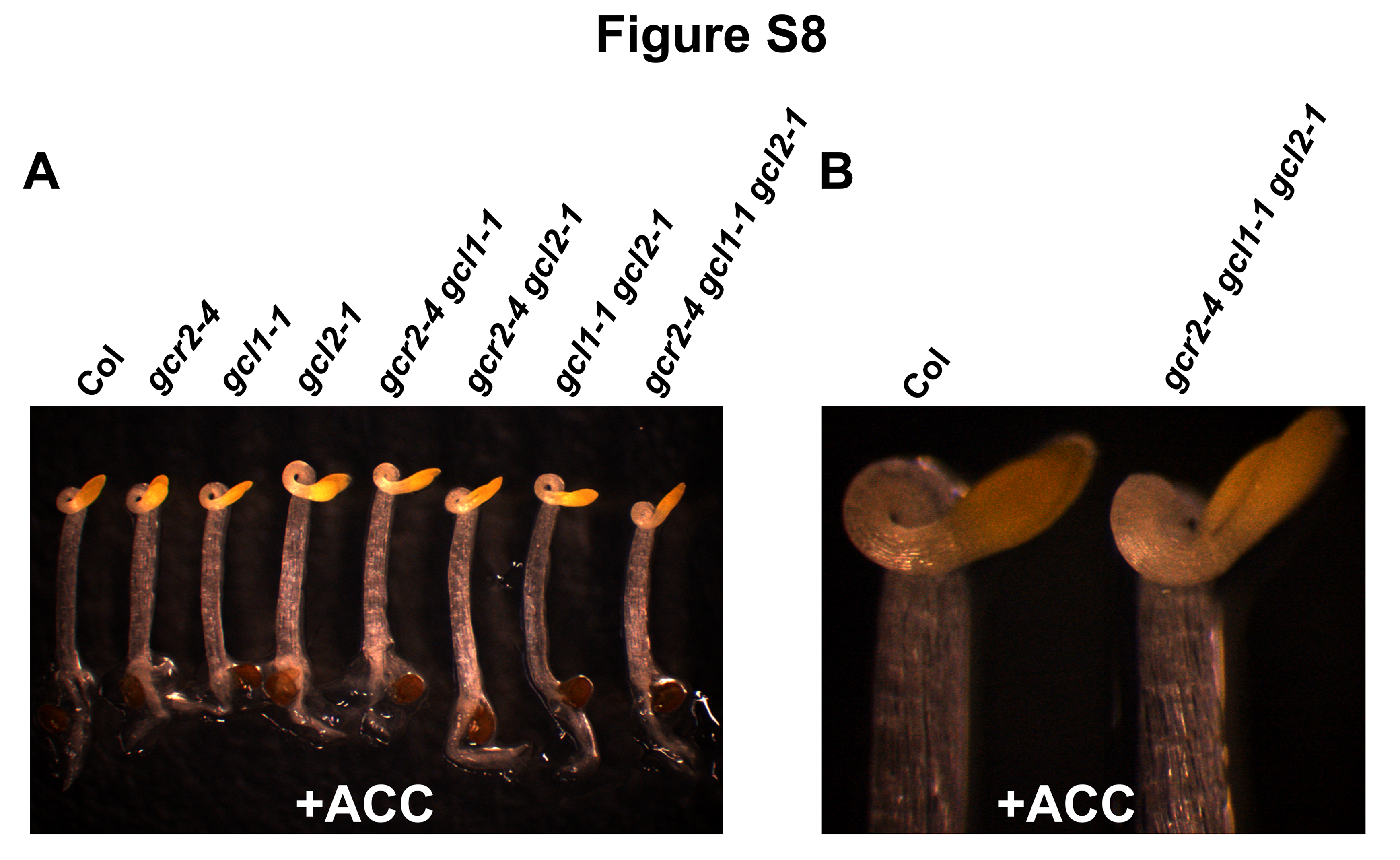

Supplement: Figure S8 — Sensitivities of gcr2, gcl1 and gcl2 single, double and triple mutants to ethylene in the triple response assay. Surface-sterilized wild-type and mutant seeds were directly sown on MS/G medium with or without 10 µM 1-aminocyclopropane-1-carboxylic acid (ACC), an ethylene precursor, and cold-treated at 4°C in dark for 2 days. Then, imbibed seeds were transferred to 23°C in dark. Shown are 3 d-old, dark-grown seedlings in the presence of 10 µM ACC. (A) The phenotype of gcr2, gcl1 and gcl2 single, double and triple mutants in response to 10 µM ACC. (B) The hook region of wild-type Col (left) and gcr2 gcl1 gcl2 triple mutant (right) in the presence of 10 µM ACC. (5.30 MB TIF) [file pone.0002982.s008.tif]
